# Sensor Fusion Method for Object Detection and Distance Estimation in Assisted Driving Applications

**DOI:** 10.3390/s24247895

**Published:** 2024-12-10

**Authors:** Stefano Favelli, Meng Xie, Andrea Tonoli

**Affiliations:** 1 Center for Automotive Research and Sustainable Mobility (CARS@PoliTO), Politecnico di Torino, 10129 Torino, Italy; andrea.tonoli@polito.it; 2 Dipartimento di Ingegneria Meccanica e Aerospaziale (DIMEAS), Politecnico di Torino, 10129 Torino, Italy; meng.xie@polito.it

**Keywords:** ADAS, environment perception, object detection, sensor fusion, camera, LiDAR, ROS

## Abstract

The fusion of multiple sensors’ data in real-time is a crucial process for autonomous and assisted driving, where high-level controllers need classification of objects in the surroundings and estimation of relative positions. This paper presents an open-source framework to estimate the distance between a vehicle equipped with sensors and different road objects on its path using the fusion of data from cameras, radars, and LiDARs. The target application is an Advanced Driving Assistance System (ADAS) that benefits from the integration of the sensors’ attributes to plan the vehicle’s speed according to real-time road occupation and distance from obstacles. Based on geometrical projection, a low-level sensor fusion approach is proposed to map 3D point clouds into 2D camera images. The fusion information is used to estimate the distance of objects detected and labeled by a Yolov7 detector. The open-source pipeline implemented in ROS consists of a sensors’ calibration method, a Yolov7 detector, 3D point cloud downsampling and clustering, and finally a 3D-to-2D transformation between the reference frames. The goal of the pipeline is to perform data association and estimate the distance of the identified road objects. The accuracy and performance are evaluated in real-world urban scenarios with commercial hardware. The pipeline running on an embedded Nvidia Jetson AGX achieves good accuracy on object identification and distance estimation, running at 5 Hz. The proposed framework introduces a flexible and resource-efficient method for data association from common automotive sensors and proves to be a promising solution for enabling effective environment perception ability for assisted driving.

## 1. Introduction

Environment perception is a fundamental task for every control pipeline related to assisted and autonomous driving. It constitutes the foundation layer of the architecture, being essential to ensure safe and efficient navigation and decision-making. This process involves the continuous collection and analysis of data from various sources, such as cameras, LiDARs, radars, and ultrasonic sensors [1]. Through sensor fusion and data association, sensors’ inputs are used to detect and categorize objects, recognize road signs, and assess road conditions [2]. By providing real-time situational awareness, environment perception enables intelligent vehicles to make informed decisions, including obstacle avoidance, speed control, and path planning, thereby ensuring safety, comfort, and eventually fuel economy [3].

The development of new Advanced Driver Assistance Systems (ADASs) has played a major role in both academic and automotive industry research activities in recent years [4]. The proposal of algorithms intended to enhance drivers and passengers’ experiences in vehicles has represented an important catalyst for the adoption of novel and more advanced perception pipelines. ADASs have traditionally been synonymous with safety and comfort applications [5], exploiting position sensors such as radars and ultrasonics to improve driver assistance and occupant well-being. However, the paradigm is shifting, with an increasing emphasis on leveraging new technologies and more advanced sensing techniques to boost overall vehicle usage and energy efficiency. This transition pushes the development of new hardware as well as new sensor fusion techniques that extend beyond their conventional use. While safety and comfort remain paramount, the demand for eco-driving solutions has become an essential component of modern mobility [6]. The ADAS is at the forefront of this evolution, offering a bridge between safety, comfort, and sustainability.

The efficient fusion of multiple sensors’ data in real-time remains a crucial process to solve the perception problem in the current ADAS, where the computational resources are scarce and the cost is a major constraint. The high-level controllers need a large amount of information on the surrounding environment to plan their next control action effectively. The focus of ADAS is to assist the driver in making choices during common road scenarios. Classifying objects in the surroundings of the vehicle and estimating their relative positions are important tasks in reconstructing common road scenarios.

This paper presents an open-source framework to estimate the distance between a vehicle equipped with perception sensors and different road objects on its path, using the association and fusion of information from different data sources. Although the solution to the perception problem is well known in the literature, it is difficult to find openly available and scalable frameworks to deploy sensor fusion from multiple sources on commercially available hardware. The purpose of this work is to provide an easily scalable and customizable method to deploy a complete perception pipeline on low-cost hardware, addressing the common challenges of embedded software development in automotive. The solution is meant to be used as a support for rapid prototyping of high-level control logics and ADASs, such as the application of vehicle dynamics controllers, speed planning algorithms, and eco-driving techniques, which can be categorized under the name of Eco-Driving Assistance Systems (EDASs).

The final results of the proposed method have been tested in real-world scenarios on commercial hardware running open-source software packages. The pipeline presented can be employed straightforwardly to solve the perception problem for ADASs or EDASs, which aim to leverage information from sensor fusion to improve the situational awareness of the algorithm on the road. In the case of the application, the sensor fusion method is coupled with a real-time rapid prototyping electronic control unit (ECU) to control the longitudinal motion of a light-duty commercial vehicle to minimize its energy consumption in urban environments.

The remainder of this work is organized as follows: Section 2 describes the state-of-the-art sensor fusion methods and related techniques used for object distance estimation. Section 3 presents the proposed methodology to fuse the data acquired by a camera and a LiDAR and its software implementation. Section 4 describes the setup of the experiments and methods used to evaluate the effectiveness of the approach. Finally, Section 5 discusses the experimental results based on the real-world scenarios of the application presented and contains the authors’ conclusions.

## 2. Related Work

High-resolution cameras are typically used to determine the shape and texture of complex objects, such as road signs and traffic signals [7]. Although computer vision algorithms have made some progress in visual 3D detection, the task remains challenging due to a lack of accurate depth information [8].

LiDAR is another commonly used sensor that can accurately calculate the object’s distance relative to the sensor, creating a detailed point cloud representation of the environment. However, one notable drawback of LiDAR is that its effectiveness diminishes at greater ranges, resulting in sparser point clouds and decreased object detection capabilities. This limitation is critical in scenarios where long-range detection is necessary, as it can compromise the system’s ability to respond to distant obstacles effectively.

Camera and LiDAR systems can also be adversely affected by weather conditions such as heavy rain, snow, or fog [9]. Such environmental challenges not only heighten the risks associated with autonomous driving but also pose significant hurdles to the advancement of related technologies. Ensuring safety and reliability in varied weather conditions is essential for the widespread adoption of autonomous vehicles.

To achieve high-level autonomous driving capabilities, multi-modal fusion of sensor data is increasingly recognized as a necessity [10,11,12,13]. Fused data is often collected from various sources, including 3D radar, cameras, LiDAR, and 4D radar, because each sensor type contributes with unique advantages. For instance, while LiDAR provides high accuracy in detailed surroundings, 4D radar offers extended detection ranges (exceeding 500 m) and better performance under challenging conditions.

The fusion of these diverse data streams allows autonomous systems to leverage the strengths of each sensor, compensating for their weaknesses. By combining the precise distance information from LiDAR with the comprehensive environmental understanding from cameras and the long-range capabilities of 4D radar, assisted driving systems can also benefit from the integration of different data streams. This multi-faceted perspective is critical for making real-time decisions and ensuring the safety of passengers, pedestrians, and other road users.

Despite the significant advancements in sensor technologies and multi-modal fusion for autonomous driving, several open points and research gaps remain. One major area that requires further exploration is the improvement of the algorithms for effective data integration from diverse sensors. Current methods struggle with the challenges posed by sensor discrepancies, such as varying resolutions, noise levels, and operational ranges. Additionally, there is a need for robust perception techniques that can effectively handle the complexities of real-world scenarios, including dynamic environments with rapidly changing conditions such as urban environments. Another critical gap is the development of standardized benchmarks and evaluation metrics to assess the performance of perception systems, which will be only partly discussed in this paper. The objective here is to assess the performance of a rather simple but flexible setup specifically in urban scenarios and provide an open platform for generalized development of fusion algorithms.

## 3. Methodology

The proposed sensor fusion approach is based on a calibrated camera-LiDAR sensor set, a properly trained object detector, and a ROS (Robot Operating System) ([14]) environment running on an embedded onboard computer. The goal is to combine sensor data to extract the detected objects’ positions so that a high-level ADAS can use it. The sensor fusion process consists of six steps: time synchronization of the data flows, preprocessing of the point cloud, distance calculation, projection of the 3D point cloud to the 2D images, distance measurement of the detected object, and output of the complete information.

### 3.1. Time Synchronization of the Data Flows

The fusion algorithm needs to subscribe to two topics: a source of point cloud data and an image with bounding boxes. The two data flows considered in the application proposed are 2D images from a camera and 3D point clouds from a LiDAR. The streams usually come at different rates: in the case of LiDAR, point cloud data is available at 10 Hz, while the object detector outputs images at a maximum rate of 5 Hz on the platform considered.

**Figure 1:**
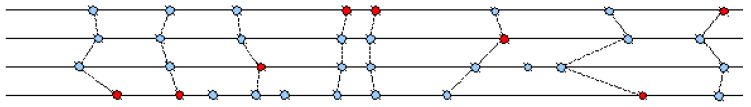
ApproximateTime policy graphical representation with four data streams. The red dots in the figure represent the pivot data points used to synchronize the different messages in one sample.

A data synchronization method is embedded in the pipeline to allow real-time fusion of the data streams. ROS provides an embedded policy-based time synchronizer in the “message_filters” library, which takes in messages of different types from multiple sources and outputs them only if it has received a message from each of those sources with the same timestamp. There is a timestamp in the header of both the data frames considered. Two policies can be adopted at this stage: the ExactTime and the ApproximateTime [15,16]. The ExactTime policy requires messages to have exactly the same timestamp in order to match, while the ApproximateTime policy uses an adaptive algorithm to match messages based on their timestamps. Since it is unrealistic for the sources considered to have frames from the two data flows at the exact same time, the ApproximateTime policy has been chosen. Its process to find the best match by allowing time differences is shown in Figure 1.

### 3.2. Preprocessing of 3D Cloud Points

The core of the fusion process is a callback function that uses as input the point cloud data of the *sensor_msgs*::*PointCloud*2 message type and the image data with detection information of the vision_msgs::Detection2DArray message type.

The PCL library [17] is used to process the point cloud data, and OpenCV [18] to process the image data. The first task is to convert the message in *sensor_msgs*::*PointCloud*2 data type to the PointCloud<pcl::PointXYZRGB> data type in PCL and apply a filter on the lateral direction to limit the field of view (FoV) to 10 m on each side. The purpose is to focus on detecting the distance only of the objects in front of the vehicle and in the adjacent lanes.

#### 3.2.1. Point Cloud Filtering and Downsampling

The second task is to filter out the points belonging to the ground to reduce the size of the point cloud and lower the computational burden of the following steps. The points on the ground account for a considerable portion of the cloud, and they can affect the accuracy of the clustering task. Many methods are used in the literature for ground filtering, such as RANSAC [19], morphological approaches [20], or machine learning-based methods [21]. However, those methods are complex and resource-intensive, which is against the purpose of this preliminary step. To increase accuracy in the clustering step, a straightforward approach has been selected: a threshold on the z-axis is set to filter out points on the ground according to the position of the LiDAR relative to the ground. The pcl::PassThrough function is used to implement this operation.

The adopted method is the well-known voxel grid downsampling [22]. The pcl::VoxelGrid function is applied to realize the voxel grid downsampling. Despite being a little slower, it has proven to be more accurate, so it uses the centroid as the representative point. The voxel grid leaf size is set to 10 cm, according to the planned application scenario where the common detection distance is between 5 and 20 m. Experiments with sizes in the range of 5 to 30 cm have proven that the selected size retains the best trade-off. This process effectively reduces the number of points by preserving the overall distribution and shape of the original point cloud.

#### 3.2.2. Point Cloud Clustering and Data Association

The next step involves cluster extraction from the downsampled point cloud to enhance detection capabilities. Challenges such as object overlap, background noise, and hardware misalignment can degrade data association. By downsampling, distant objects become sparser while closer objects remain prominent. Proper clustering parameters help filter out background noise, ensuring that detected object distances are accurately measured, even admitting projection deviations.

In the proposed approach, extraction of the Euclidean clusters is performed with the pcl::EuclideanClusterExtraction class. The class provides various parameters that can be adjusted to control the clustering behavior, including the minimum and maximum cluster sizes, the distance threshold for considering points as neighbors, and the search method to use. The first step is to define a search method for the extraction, and PCL provides three main search methods, namely KdTree [23], Octree [24], and FlannSearch [25].

In the considered application, KdTree is the most suitable for a fast and efficient nearest-neighbor search. Its main parameters (cluster tolerance and min–max cluster sizes) have been chosen accordingly. The cluster tolerance is the distance threshold used to specify the maximum distance between two points for them to be considered belonging to the same cluster. This parameter has a significant impact on the quality of clustering, as a larger tolerance value allows greater distances between points, resulting in larger clusters, and vice versa. In the considered application, the tolerance is set to 30 cm. With a chosen voxel size of 10 cm during downsampling, it is reasonable to assume that for the two adjacent voxels in the diagonal direction, the maximum distance between the farthest points is smaller than 35 cm.

The other two parameters are minimum and maximum cluster size, which represent the minimum and maximum number of points required for a cluster to be considered valid. The values chosen are 50 for the minimum and 20,000 for the maximum because the main target for the downsampling and clustering is to filter out the noisy background. As it is reported in Section 4.1.1, there are at most 64,000 points (32 rows × 2000 columns) in each frame of the point cloud considered. By limiting the FoV to 120° horizontally, the number of points left after the preprocessing is around 1/3 of the total points. According to our experience in processing the point cloud of the HESAI PandarXT-32, for the largest object encountered on the road (i.e., a bus), the number of points in the cloud does not exceed 20,000 points, and for the smallest object detected (i.e., a pedestrian), the number of points in the cloud is typically more than 50 to ensure a meaningful detection. So, the pair of parameters mentioned above is safe to ensure acceptable performance.

The set of parameters presented in this section and reported in Table 1 has proven to be the most reliable during the experimental tests of the pipeline, as discussed in Section 4.

### 3.3. Multiple Objects’ Distance Estimation

There are different ways to define the distance between a vehicle and surrounding objects, each with its advantages and considerations. One approach is to leverage point cloud data to calculate the three-dimensional Euclidean distance. By working in three-dimensional space, an accurate measure of the physical distance between the 3D sensor and the object is obtained, as reported in (1).

(1) $$d=\sqrt{{\left(x_{P}-x_{O}\right)}^{2}+{\left(y_{P}-y_{O}\right)}^{2}+{\left(z_{P}-z_{O}\right)}^{2}}$$

where the following is true:

- ($x_{O}$, $y_{O}$, $z_{O}$) are the 3D coordinates of the origin of the reference frame of the sensor.
- ($x_{P}$, $y_{P}$, $z_{P}$) are the 3D coordinates of the object represented in the reference frame of the sensor.

Another viable option is to directly use the distance information along the y-axis to represent the object’s distance in front of the vehicle, i.e., the longitudinal distance. This approach assumes that the vehicle’s forward direction aligns with the y-axis of the LiDAR. By considering only the longitudinal distance, the estimation is simplified and the problem’s dimensionality is reduced. This method can be particularly useful in certain scenarios where the precise three-dimensional location of objects might not be necessary, i.e., for purely longitudinal vehicle dynamics control, as in the case of the application.

#### 3.3.1. 3D to 2D Projection

The projection of the 3D cloud points onto 2D Yolov7 detection images is the core of the proposed algorithm. This operation aims at assigning the LiDAR points to the corresponding objects in the camera images. To get the LiDAR and the camera to work together, it is crucial to perform the joint calibration between the sensors and work in a common coordinate system. The joint calibration typically involves determining the relative position and orientation between the sensors [26], which is described by the extrinsic parameters.

The model of the image frame considered in this work during the projection procedure is the distortion-free projective transformation given by the pinhole camera model [27]. By applying the model, the roto-translation into a 3D coordinate frame is described by (2)

(2) $$p_{C}=A\left[\begin{array}{c} R|t \end{array}\right]P_{L}$$

where the following is true:

- $p_{C}$ is a 2D pixel in the image plane in the camera coordinate system [u;v;1];
- *A* is the matrix representing the intrinsic parameters of the camera;
- [R|t] is the roto-translation matrix representing the extrinsic parameters;
- $P_{L}$ is a 3D point expressed in the LiDAR coordinate system $\left[X_{L};Y_{L};Z_{L};1\right]$.

The intrinsic parameter matrix *A* is used to project the 3D points in the camera coordinate system $P_{C}=\left[X_{C};Y_{C};Z_{C}\right]$ to 2D pixel coordinate system $p_{C}=\left[u;v;1\right]$:

(3) $$p_{C}=AP_{C}$$

*A* is a 3×3 matrix composed of the focal lengths $f_{x}$ and $f_{y}$, which are expressed in pixel units, and the principal point ($c_{x},c_{y}$), which is usually the image center:

(4) $$A=\left[\begin{array}{ccc} f_{x} & 0 & c_{x}\\ 0 & f_{y} & c_{y}\\ 0 & 0 & 1 \end{array}\right]$$

Therefore, the projection Equation (3) can be expressed as follows:

(5) $$\left[\begin{array}{c} u\\ v\\ 1 \end{array}\right]=\left[\begin{array}{ccc} f_{x} & 0 & c_{x}\\ 0 & f_{y} & c_{y}\\ 0 & 0 & 1 \end{array}\right]\left[\begin{array}{c} X_{C}\\ Y_{C}\\ Z_{C} \end{array}\right]$$

The intrinsic parameters do not depend on the scene represented and are provided in the camera calibration file. In the ZED2 case, matrix *A* also comprises the distortion coefficients.

The transformation matrix [R|t] in (2) represents the extrinsic parameters from the sensors’ calibration, matrix *R* and vector *t*. It is used to perform the change of basis from the LiDAR’s coordinate system *L* to the camera’s coordinate system *C*. *R* is a 3×3 rotation matrix, and *t* is a 3×1 translation vector. It is defined as follows:

(6) $$\left[\begin{array}{c} R|t \end{array}\right]=\left[\begin{array}{cccc} r_{11} & r_{12} & r_{13} & t_{x}\\ r_{21} & r_{22} & r_{23} & t_{y}\\ r_{31} & r_{32} & r_{33} & t_{z} \end{array}\right]$$

From this definition, a 3D point $P_{L}=\left[X_{L};Y_{L};Z_{L};1\right]$ detected by the LiDAR can be represented in the camera frame as $P_{C}=\left[X_{C};Y_{C};Z_{C}\right]$ applying [R|t] as follows:

(7) $$\left[\begin{array}{c} X_{C}\\ Y_{C}\\ Z_{C} \end{array}\right]=\left[\begin{array}{cccc} r_{11} & r_{12} & r_{13} & t_{x}\\ r_{21} & r_{22} & r_{23} & t_{y}\\ r_{31} & r_{32} & r_{33} & t_{z} \end{array}\right]\left[\begin{array}{c} X_{L}\\ Y_{L}\\ Z_{L}\\ 1 \end{array}\right]$$

Therefore, when the parameters are combined, Equation (2) can be rewritten as follows:

(8) $$\left[\begin{array}{c} u\\ v\\ 1 \end{array}\right]=\left[\begin{array}{ccc} f_{x} & 0 & c_{x}\\ 0 & f_{y} & c_{y}\\ 0 & 0 & 1 \end{array}\right]\left[\begin{array}{cccc} r_{11} & r_{12} & r_{13} & t_{x}\\ r_{21} & r_{22} & r_{23} & t_{y}\\ r_{31} & r_{32} & r_{33} & t_{z} \end{array}\right]\left[\begin{array}{c} X_{L}\\ Y_{L}\\ Z_{L}\\ 1 \end{array}\right]$$

The objective of calibration is to acquire the extrinsic parameters *R* and *t* in a consistent and repeatable fashion [28]. Both are very sensitive to noise during the feature extraction process, as small errors in rotation or translation estimation can greatly affect the usability of the calibration result. Tsai et al. [29] proposed a well-designed method to perform the calibration using multiple sets of poses on a chessboard to obtain a robust estimate of the calibration parameters with their uncertainty. This method was adopted to calibrate the proposed LiDAR-camera system; the results of the calibration are reported in Section 4.2.1.

The cv::projectPoints function is employed to execute the projection process. The input to the function is an array of 3D LiDAR points, and the output yields an array of corresponding 2D image points, specifically the pixels within the Yolov7 detection image. Each pixel corresponds to a LiDAR point in the input data.

#### 3.3.2. Distance Measurement Association with Detected Objects

After the point cloud data is projected, the detected objects are evaluated based on their bounding boxes, which contain information about their class and dimensions. An iterative method is used to accurately identify whether projected 3D points fall within the bounding boxes. Given the diverse shapes of detected objects, corners of bounding boxes can lead to background points being mistakenly included. To mitigate this problem, the method proposed applies a re-scale factor to both the height and width of the bounding box, effectively shrinking it, which allows the algorithm to focus on the points around the center of the object. In the application presented, a re-scale factor of 90% is already capable of obtaining satisfactory behavior.

Once points within the adjusted bounding box are identified, their distances are extracted from a corresponding vector. Two methods for representing the relative distance are proposed: the minimum distance and the average distance. However, outliers can skew the average distance calculations, so a truncated mean method is implemented to ignore the highest 10% of distances. The minimum distance reflects the closest point to the ego vehicle, while the average distance provides a broader context of distances. By comparing these two metrics, the system aims to align more closely with the driver’s perception of distance, considering that the sensor’s FoV differs from the driver’s. This evaluation enhances the usability and safety of the distance measurement method, making it more intuitive for real-world driving scenarios.

#### 3.3.3. Output of the Detection Information

The last step of the algorithm proposed is to output any necessary information according to the different requirements of the upstream ADAS control logic at different development stages. At the testing stage, the algorithm is set to publish point cloud data after the downsampling and clustering to tune their parameters and visualize the projected LiDAR points on the image to check the projection’s performance. Detection information is also necessary at this stage, such as object class, confidence score, and distance information, and saving the information for post-processing.

**Figure 2:**
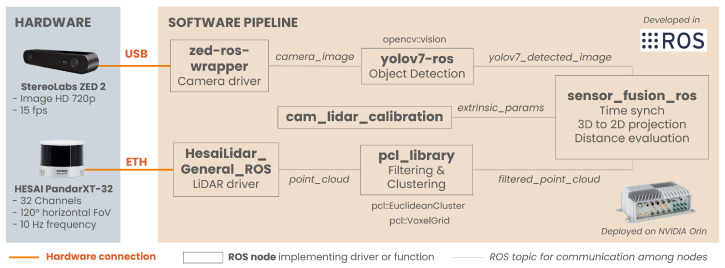
Overview of the sensor fusion pipeline with the sensors proposed for the experiments.

The overview of the sensor fusion pipeline proposed is presented in Figure 2. The code of the projection method as well as the complete pipeline used for testing are available at https://github.com/stefavpolito/perception-MOST (accessed on 21 November 2024).

## 4. Experimental Results

In this section, the experimental setup used to verify the performance of the fusion method is presented. The definition of hardware setup is presented first, and then the pipeline deployment from environment preparation to training of the object detector is addressed. The second part presents the validation tests performed on different driving scenarios to assess real-time performance and impact on the final application.

### 4.1. Experimental Vehicle Setup

#### 4.1.1. Hardware Selection and Algorithm Deployment

The onboard workstation is the AI Vehicle Computer RSL A3 from Syslogic AG (Baden, Switzerland), combining an Jetson AGX Xavier OEM module from NVIDIA Corp (Santa Clara, CA, USA) with an embedded aluminum housing resistant to shock and vibration. The operating system is NVIDIA Linux for Tegra (L4T 35.1.0), Ubuntu 20.04, JetPack 5.0.2, and ROS Noetic. ROS [14] has been chosen to ensure compatibility of various dependencies required by the detector and the drivers of the LiDAR and camera sensors.

**Figure 3:**
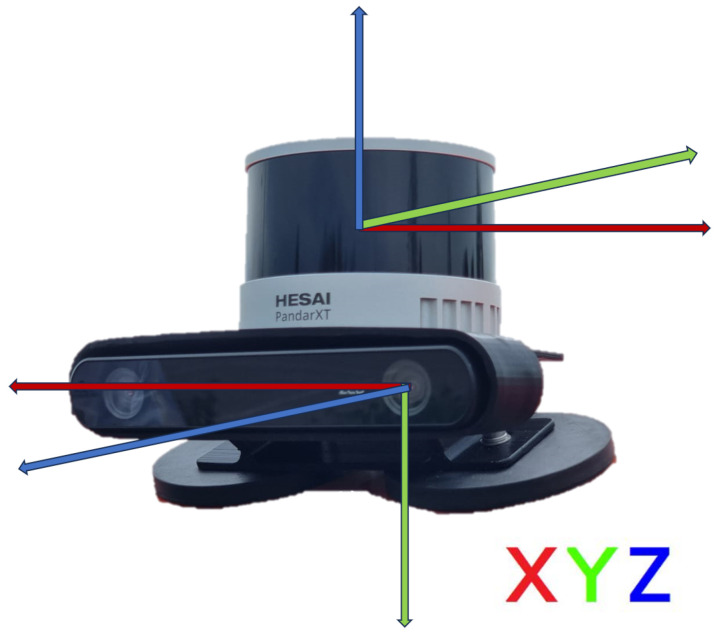
Hesai PandarXT-32 LiDAR and Stereolabs ZED2 camera integrated sensors’ setup.

**Figure 4:**
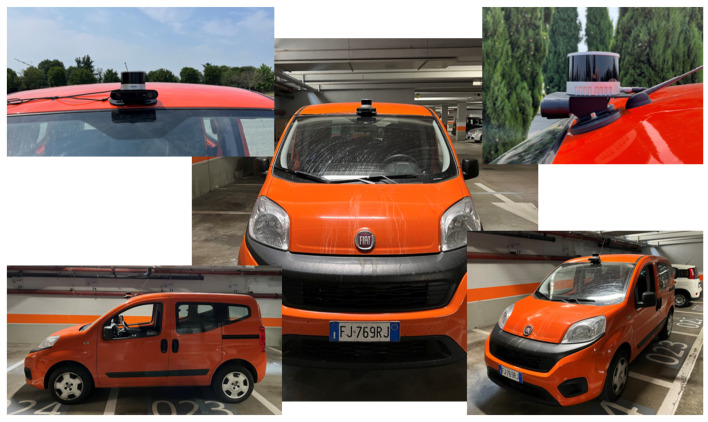
LiDAR and camera mounting position on the vehicle used for on-road data acquisition.

The sensors’ setup considered during the experimental validation consists of a camera for 2D images and a LiDAR as a source of 3D point clouds. The camera adopted is a ZED2 Stereocamera from Stereolabs Inc (San Francisco, CA, USA) that provides a 2.2k resolution video output and an FoV of 120°. The LiDAR is a PandarXT-32 from Hesai Photonics Technology Co., Ltd (Shanghai, China), featuring 32 channels with 1° of vertical resolution and horizontal FoV limited to 120°. To assess the viability of installing the sensor set on an actual vehicle, an integrated LiDAR-camera support has been designed. The configuration shown in Figure 3 has been mounted above the windshield as depicted in Figure 4.

#### 4.1.2. Training of the Object Detector Yolo v7

According to the requirements of the considered application, there are 26 classes of objects that need to be detected, including vehicles, traffic light status, speed limits, traffic signs that might affect ego speed, and other objects such as pedestrians and bikes. The detailed classes are listed as Table 2.

A custom dataset has been prepared and used to train the neural network. Many annotated datasets for autonomous driving research can be found in the literature, but their annotations do not meet all the specifications required. For this reason, the images from these datasets have been used and manually annotated using the Yolo_mark tool provided by Alexey Bochkovskiy [30], which is a graphical user interface (GUI) for marking bounding boxes of objects in images for training Yolo neural networks.

The total selection is of about 3 thousand images to train the Yolov7, which contains 1451 images from BDD100K [31], 99 images from The German Traffic Sign Recognition Benchmark (GTSRB) [32], 706 images from The German Traffic Sign Detection Benchmark (GTSDB) [33], and 852 images from a road sign detection database. The parameters used to train the Yolo v7 on a local workstation are listed in Table 3. The labeled dataset is available at github.com/stefavpolito/yolo-automotive-dataset (accessed on 21 November 2024).

### 4.2. Sensor Fusion Method Validation

#### 4.2.1. Calibration of Sensor Set

**Figure 5:**
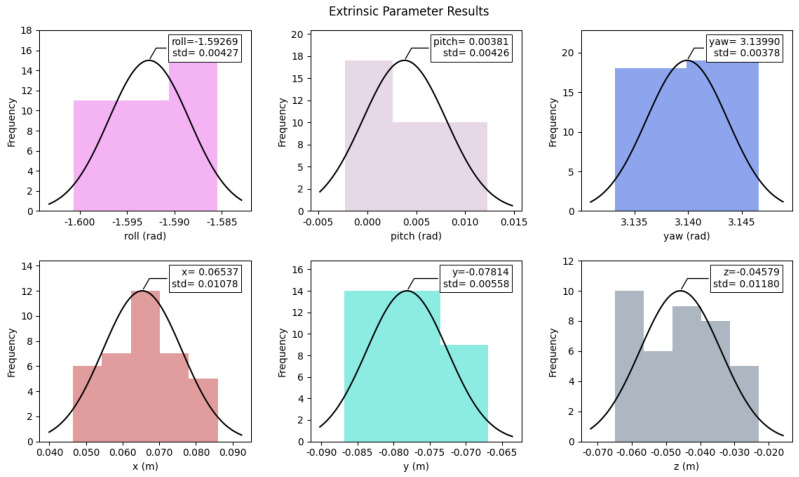
Extrinsic parameters with standard deviation from the calibration procedure [29].

To validate the sensor fusion method on a real test vehicle, a calibration step is needed to retrieve the extrinsic parameters of the 3D-to-2D projection. According to [29], the calibration procedure employed 33 poses captured with a chessboard with 7 × 5 inner vertices with a 90 mm square length and a board dimension of 565 × 793 mm. The output parameters are tightly linked to the mounting of the sensors and are reported as the peaks of the Gaussian fit curve of Figure 5, where the histogram of their accuracy is also reported.

The output calibration parameters in Table 4 describe the transformation from the camera frame to the LiDAR frame, which means that the camera coordinate system is the parent coordinate system, and that is the rule defined in the OpenCV library. However, in the fusion algorithm, the rotation vector required by OpenCV is in Rodrigues rotation representation, while the rotation vector given by the calibration algorithm is in Euler angle representation. The additional transformation required has been employed to compute the final rotation vector [0.0061, 2.2445, −2.1959] and translation vector [0.0654, −0.0781, −0.0458].

#### 4.2.2. Assessment of Distance Measurement Accuracy

**Figure 6:**
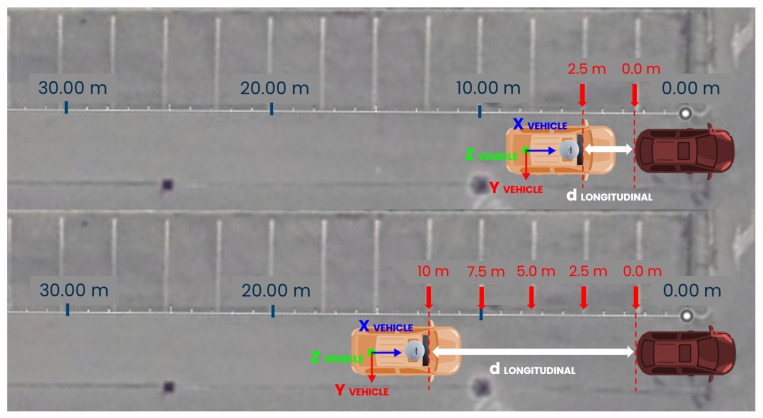
Distance measure evaluation in parking lot scenario with target vehicle.

The evaluation of the distance measure accuracy is conducted in controlled conditions on two tests. The first scenario is an empty parking lot with standard 2.5-meter-wide parking spaces. As shown in Figure 6, a target vehicle is parked in a fixed position, while the test vehicle starts 2.5 m behind it. The test vehicle moves backward, increasing the distance by 2.5 m at each step until the target is out of the Yolov7 detection range. This setup allows comparison between measurements obtained from the sensor fusion algorithm and a ground truth distance measurement, verifying the system’s functionality in real applications.

**Figure 7:**
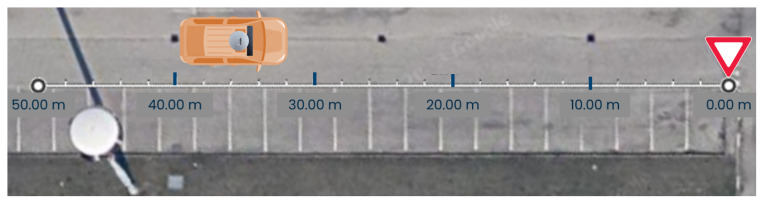
Distance measure evaluation in parking lot scenario with yield sign target.

The second test follows a similar procedure but uses a yield sign as the target, as shown in Figure 7. The test vehicle approaches the sign to determine the maximum distance at which the system can recognize it. This test also compares the Euclidean distance representation with the longitudinal distance representation, highlighting the importance of choosing the most suitable distance representation to use LiDAR’s precision effectively.

**Figure 8:**
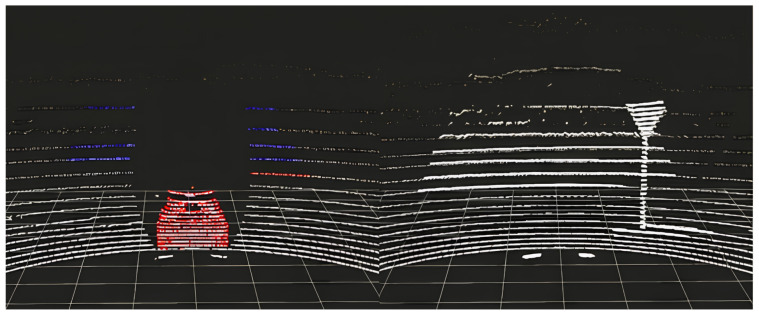
Parking lot scenario point cloud visualization (**left**: vehicle, **right**: yield sign).

The LiDAR point cloud representations of the two tests are reported in Figure 8.

**Figure 9:**
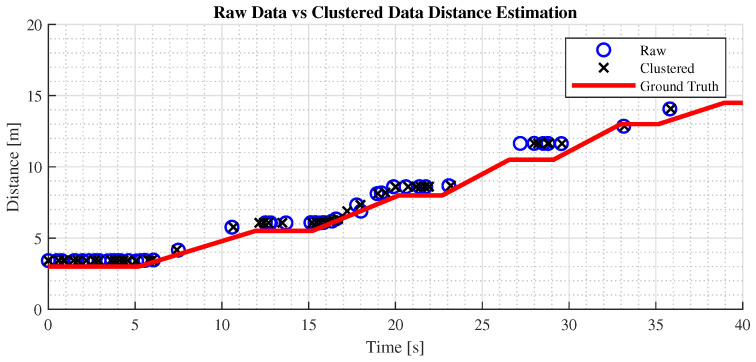
Comparison of distance estimation methods on vehicle detection in parking lot.

**Figure 10:**
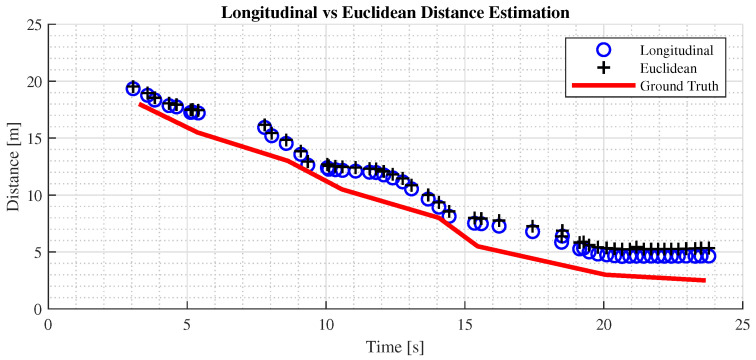
Comparison of distance estimation methods on yield sign detection in parking lot.

In Figure 9, it can be observed that the measured distances closely align with the ground truth, indicating that the LiDAR’s ranging capability is reliable and precise when measuring a single target directly in front of the vehicle, with minor disparities between the two distance representations. However, as shown in Figure 10, in the second test using the yield sign, the difference between the distance representations becomes more pronounced.

**Figure 11:**
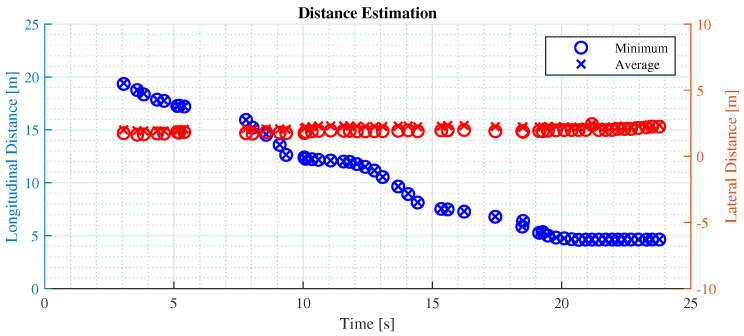
Longitudinal and lateral distance estimation performance. The blue data points in the figure represent the longitudinal distance estimation, while the red points represent the lateral one.

This disparity arises because the Euclidean method takes into account the height (*z*-axis) and lateral distance (*x*-axis) of the detected object, as depicted in Figure 11. This test proved the hypothesis in Section 3.3, and it can be concluded that in common driving scenarios, the longitudinal distance representation is more aligned with the driver’s perception of distance.

### 4.3. Distance Estimation Method Validation

#### 4.3.1. Assessment of the Stability of Object Detection and Distance Measurement

Also in this case, two tests have been implemented to assess the stability of the data association during common driving, namely in a car-following scenario in urban areas.

**Figure 12:**
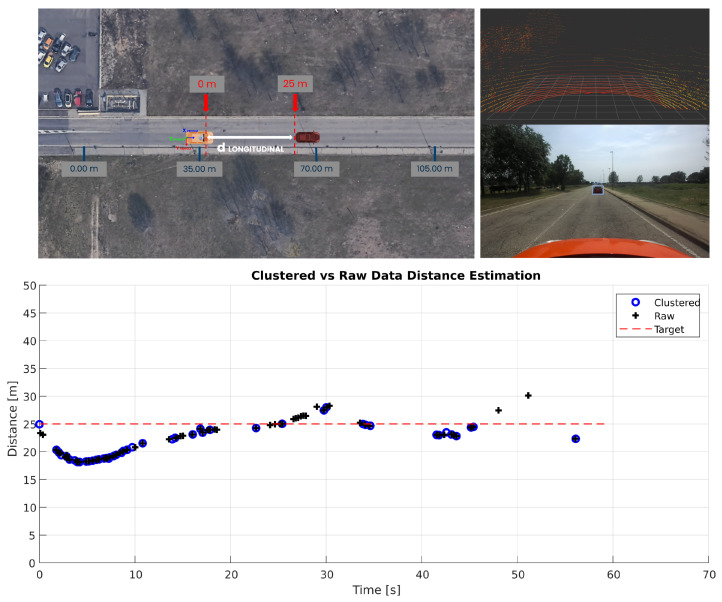
Test performed on a car-following scenario on a suburban road.

The first test is set on an empty suburban road where a target vehicle gradually moves ahead until it is no longer recognized. The test vehicle pursues the target until it re-establishes a fixed following distance, as shown in Figure 12. The test aims to benchmark the effectiveness of raw versus preprocessed data, using as metrics the maximum effective detection distance and root mean square error (RMSE) of the distance measurements, with longitudinal measurement as the primary distance representation.

Figure 12 shows that the maximum stable detection distance for clustered data is 25 m, while raw data can reach 30 m, aligning with drivers’ focus in urban environments. This detection range meets the ADAS requirements for this scenario. Additionally, both preprocessed and raw data yield consistent, reliable distance measurements, demonstrating the fusion algorithm’s effectiveness in maintaining accuracy during car-following.

**Figure 13:**
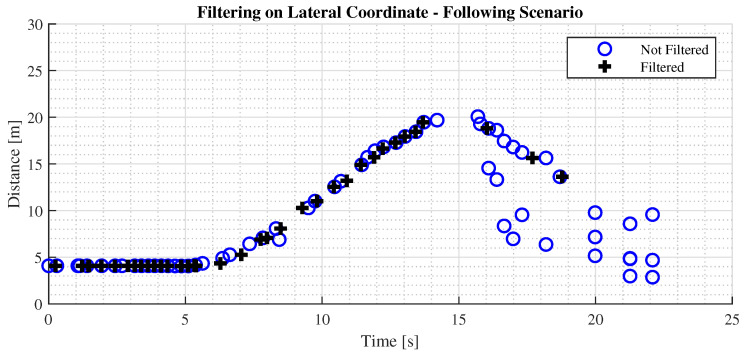
Detection in the following scenario of filtering on the lateral coordinate.

Furthermore, a filter on the lateral coordinate has been implemented to restrict the FoV and focus on the current lane. Figure 13 presents a comparison of the performance on the final distance estimate.

**Figure 14:**
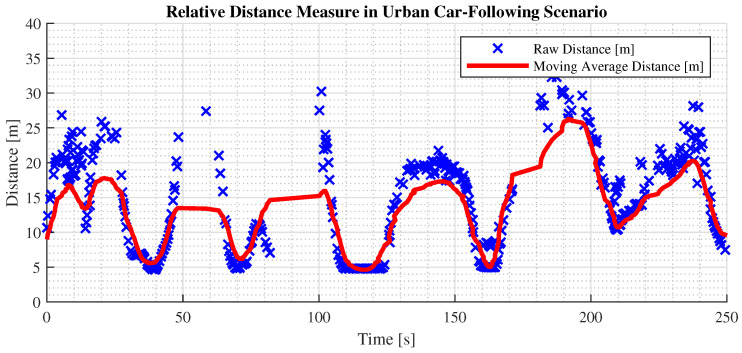
Relative distance acquisition: raw data in blue and filtered data in red.

The second test focuses on the stability of the measurement in a typical urban driving scenario, where a vehicle in front is followed and several traffic lights and signs are encountered. The distance measurements captured provide valuable insights into the system’s performance. As shown in Figure 14, the minimum distance recorded is 5 m, indicating that a safe distance from the vehicle in front is guaranteed when coming to a stop. The maximum distance observed is 30 m, representing the system’s maximum ranging capability.

The stability and the repeatability of the measured distances are crucial aspects of an ADAS, as they directly impact the system’s ability to make informed decisions. An example is the usage of distance information to provide timely warnings to the driver in applications such as Adaptive Cruise Control (ACC) enhanced with emergency brake functionality.

#### 4.3.2. Performance in the Detection of Multiple Objects and Distance Estimation

The next tests aim to assess the system’s performance in detecting multiple objects and accurately measuring their relative distances. The experiments have been conducted in three different locations, each representing real-world driving scenarios that can occur during the normal operation of vehicles in urban conditions:

- **Single-lane road in neighborhood**: the scenario involves a typical neighborhood with parked cars, pedestrians, bicycles, and traffic signs, aiming to assess the reliability in detecting and measuring distances in a complex environment.
- **Two-lane road without traffic light**: the tests were conducted on a two-lane road with low traffic, featuring pedestrians and vehicles, to evaluate the performance in a realistic urban driving environment without traffic lights.
- **Three-lane road with traffic light**: the scenario involved approaching a traffic light on a three-lane road with lane switching. It aimed to assess the distance measurement and traffic light detection in dynamic conditions.

The criteria proposed to evaluate the performance of the fusion method in the previously defined scenarios are the following:

- **Accuracy of Yolov7 detection**: Yolov7’s detection accuracy is assessed by comparing correct and false detections against the total number of objects, evaluating its reliability in real-world scenarios.
- **Distance representations**: the average distance of measured objects by class is calculated, allowing for a comparison of representations to evaluate the effectiveness in reflecting the proximity between the test vehicle and the encountered objects.
- **Valid ranging ratio**: the ratio of valid distance detections to the total detections by object class is evaluated, offering insights into the system’s ranging capability for various object classes.

#### 4.3.3. Discussion of the Results on Multiple Object Detection Scenarios

**Figure 15:**
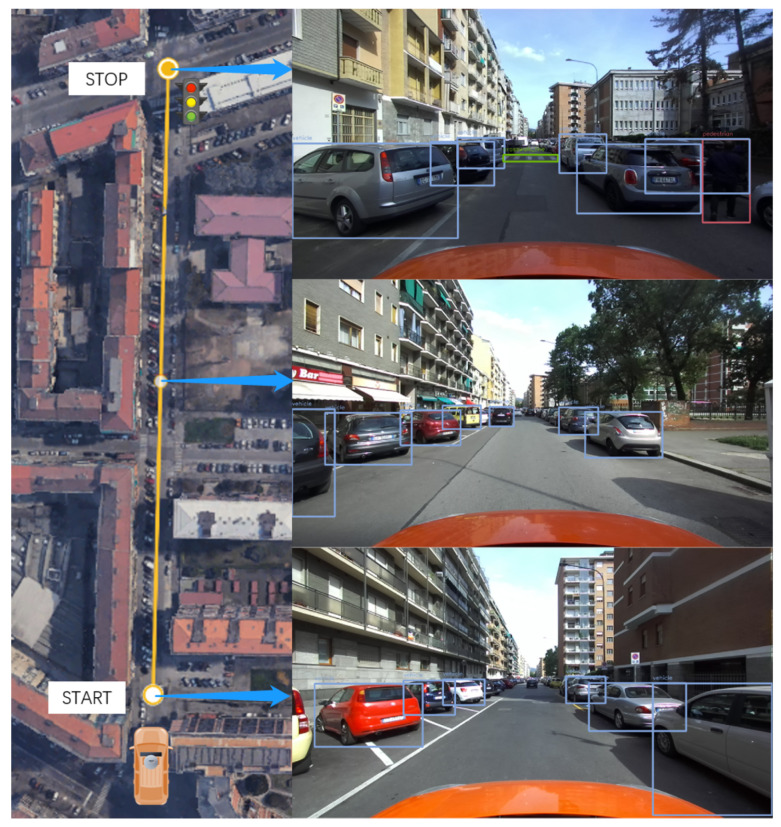
Single-lane road in neighborhood.

**Figure 16:**
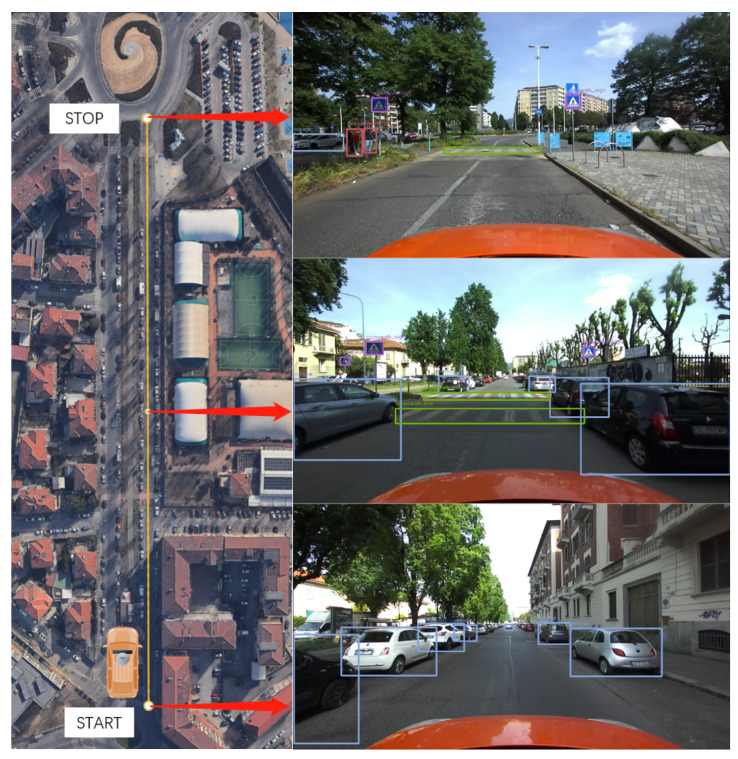
Two-lane road without traffic light.

**Figure 17:**
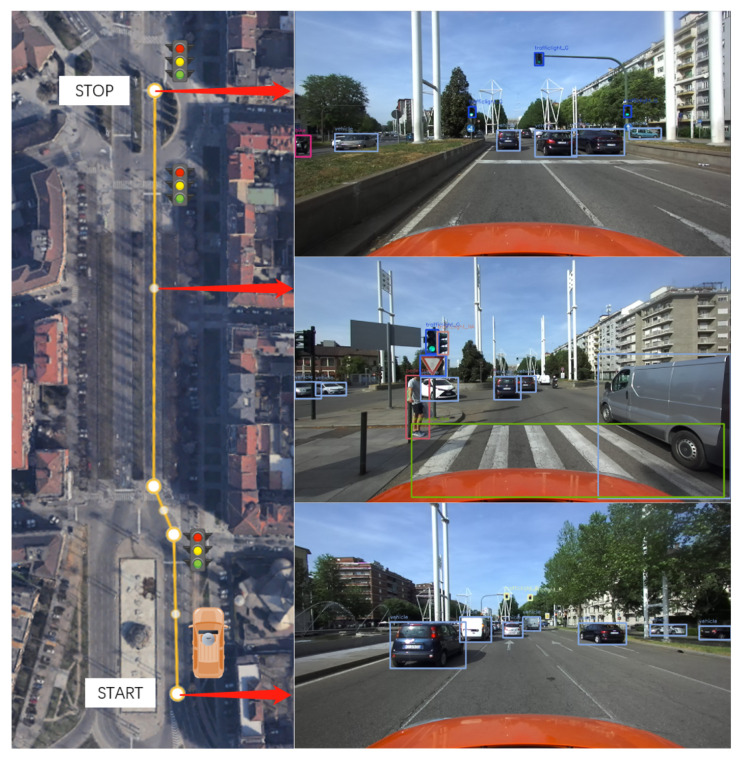
Three-lane road with traffic light.

After data acquisition and post-processing, the following results can be highlighted from the three scenarios proposed to assess performance on multi-object detection. The discussion is divided into the three scenarios presented in the previous section:

- **Single-lane road in neighborhood**: As shown in Table 5 and Figure 15, Yolov7 demonstrates high precision in detecting vehicles within a crowded environment. However, there may be instances of false detections for less usual objects. For objects with valid distance measures, the average distances obtained are found to be reasonable. However, it is worth mentioning that the measurements for traffic signs may not always be valid because of the challenge of recognizing them steadily as targets.
- **Two-lane road without traffic light**: As shown in Table 6 and Figure 16, Yolov7 performs better in a less crowded environment. The average distances are also acceptable for objects with valid distance measures. The acquisitions for traffic signs are still less reliable, even though they do not suffer the issue of misidentification.
- **Three-lane road with traffic light**: As shown in Table 7 and Figure 17, there are fewer objects in the scenario since the driving is in a traffic flow. The performance of Yolov7 is stable, but there are still false detections in the speed limit signs. The average distances are also reasonable; however, the measurements for traffic lights are not available because of the difficulty in data association.

## 5. Discussion

Through the analysis of the three urban driving scenarios mentioned in the last section, several points of discussion can be addressed. In the following, the discussion is divided between the main key performance indicators identified and the conclusions on the system’s performance are drawn.

**Accuracy of Yolov7 detection:** The current training of Yolov7 demonstrates satisfactory reliability in detecting common objects such as vehicles, traffic lights, and pedestrians. Remarkably, the detection success rate of vehicles and traffic lights, which are the main objects of interest in the application, is 100%. However, there are some instances of false detection when it comes to traffic signs. This discrepancy suggests that the current training data may not be as comprehensive or specific for accurate detection and recognition of traffic signs. To address this issue, further training of the model with additional dedicated datasets specifically focused on traffic signs is recommended.

**Distance representation:** It is observed that when an object is detected by Yolov7 and has a valid distance measurement, both the Euclidean and longitudinal representations provide reasonable and consistent distance measurements that align with the corresponding scenarios. Based on the tests conducted and the analysis performed, it can be concluded that considering the nature of driving scenarios and the need to match the driver’s distance perception, the longitudinal distance emerges as a more suitable representation in real-world driving situations.

**Valid ranging ratio:** The ranging ability of the system demonstrates variations across different classes of objects. Objects that are relatively large and have a clear line of sight from the LiDAR’s perspective, such as vehicles, bikes, and pedestrians, consistently yield valid distance measurements. For instance, in the first two scenarios where the objects are not obstructed by other objects, the valid ranging ratio exceeds 75%. This indicates that the system successfully provides valid distance measurements for a significant portion of the detected objects. However, in the third scenario, where several objects are present in parallel lanes, the valid ranging ratio is decreased to 65%. This stability in distance measurement validity can be attributed to the prominent presence and unobstructed visibility of these objects. However, for smaller objects, such as traffic lights and traffic signs, the validity of distance measurements is less reliable. As for the crosswalk lines on the road, they might pose challenges for the ground filtering function, leading to potential difficulties in obtaining valid distance measurements. Additionally, certain vehicles and pedestrians that are obstructed by other objects from the LiDAR’s perspective may also exhibit a lower likelihood of obtaining valid distance measurements. These findings indicate that the ranging ability of the system is influenced by the size, visibility, and potential obstructions of the objects being detected. Larger and more visible objects tend to yield more consistent and reliable distance measurements, while smaller or obstructed objects may present challenges in obtaining valid measurements.

**FoV limitation vs. fusion time:** From the tests conducted on the pipeline, the limitation of the FoV has significantly improved the fusion processing time. After limiting the x-axis to cover a range of 4 m on each side of the vehicle to focus on the adjacent lanes, it has been observed that there was a 50% reduction in the number of points that needed to be processed compared with the unrestricted scenario. Remarkably, in the operation, the fusion time was reduced to just 10% of the original processing time. This indicates that by applying an FoV limitation, it can reduce the number of point cloud data to be processed and significantly improve the fusion processing speed. This FoV filter allows for an effective trade-off between the system’s reliability and computational efficiency. It enables lowering the ‘min_cluster_size’ parameter to detect more clusters and improve the detection capability for distant objects while maintaining the appropriate processing speed. This filtering technique plays a crucial role in the system, providing an effective and reliable solution to enhance the detection of distant objects.

As a final remark, further validation and testing in diverse real-world scenarios are necessary to solidify these findings and ensure their general applicability.

## 6. Conclusions

With the increasing convergence of artificial intelligence and the automotive industry, the capability of vehicles to perceive their surroundings, process data in real-time, and integrate information has become a crucial aspect of an ADAS. This work aims to investigate the feasibility of meeting the ADAS requirements for the environmental awareness task in a real-world application and propose a practical solution.

**Figure 18:**
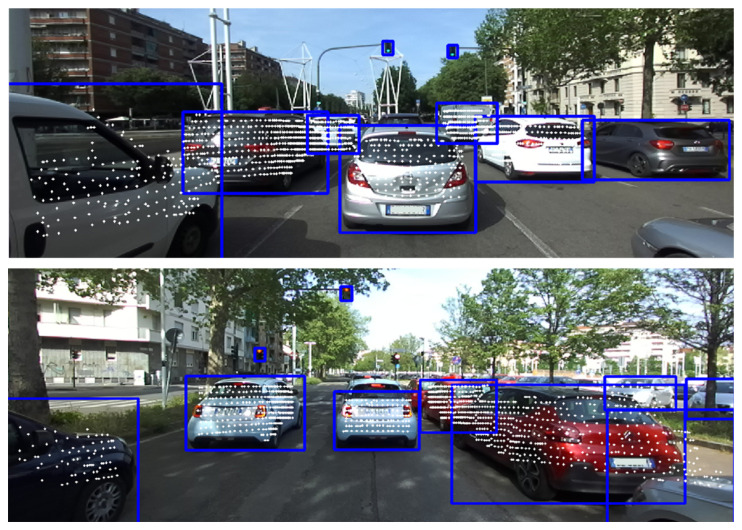
Projected 3D point clouds on 2D camera images.

Based on the experimental tests, several key conclusions can be drawn. First, the Yolov7 object detector is shown to be well-suited for real-time custom object detection tasks. Its accuracy and reliability make it a valuable choice for ADAS applications. Additionally, the preprocessing of point cloud data proves to be essential in ensuring accurate and stable detection and distance measurement in real-world driving scenarios. Although this operation can slightly reduce the effective detection distance, it is crucial to achieve reliable results in complex environments, making the trade-off between accuracy and efficiency justified in this context. Regarding distance representations, the minimum longitudinal distance has proven to be aligned with the driver’s perception of distance in real-world driving scenarios while being the most appropriate for safety distance assessment. In Figure 18 the final outcome of the pipeline in terms of 3D points projection on the camera images is shown.

This work outlines the process of exploring, designing, implementing, and evaluating a perception pipeline for an ADAS. By establishing the theoretical basis, developing hardware and software architectures, and performing real-world tests, this work aims to demonstrate the feasibility and effectiveness of the system’s environmental awareness. The data and analysis provide valuable insights for further developments, offering an opportunity to enhance performance and detection capabilities in real-world driving scenarios. On the hardware side, the integration of multiple sensors can improve redundancy and extend the detection range. This can be achieved by incorporating more advanced LiDAR sensors, radars, or additional cameras to provide a more comprehensive view of the vehicle’s surroundings. The integration of new sensors can enhance the accuracy and reliability of the perception task, and it requires a low integration effort.

From a software standpoint, further development of the fusion algorithm can be envisaged. The inclusion of an object tracking function can greatly enhance the system’s performance by enabling the tracking and prediction of actors’ movements. This functionality can improve decision-making capabilities and boost proactive responses in evolving traffic situations. Additionally, the projected image stream can be leveraged to generate an intuitive visualization for the driver, which can enhance situational awareness.

In conclusion, this work provides a solid platform for future developments. Upgrading the hardware components, improving the fusion algorithm, and incorporating advanced visualization techniques can further enhance performance, safety, and user experience. Continuous innovation and advancements in this field will contribute to the realization of more sophisticated and effective ADAS in the future.

## Figures and Tables

**Table 1:** Parameters used for point cloud preprocessing.

Parameter	Lateral Limit	Height Limit	Leaf Size	Cluster Tolerance	Min Cluster Size	Max Cluster Size
Value	5.0	−2.0	0.1	0.35	50	20,000

**Table 2:** List of labels classes used for training of Yolo v7.

ID	Class	ID	Class	ID	Class
0	vehicle	6	speedlimit_20	12	speedlimit_80
1	trafficlight_NA	7	speedlimit_30	13	speedlimit_90
2	trafficlight_G	8	speedlimit_40	14	speedlimit_100
3	trafficlight_Y	9	speedlimit_50	15	speedlimit_110
4	trafficlight_R	10	speedlimit_60	16	speedlimit_120
5	speedlimit_NA	11	speedlimit_70	17	speedlimit_130
18	yield	21	bumper_sign	24	pedestrian
19	stop	22	crosswalk_sign	25	bike
20	stop_horizontal	23	crosswalk_line		

**Table 3:** List of parameters used for training of Yolo v7.

Parameter	Workers	Batch-Size	Img	Epochs
Value	4	4	640 × 640	600

**Table 4:** Output parameters from the calibration procedure.

Parameter	Value	Unit
roll	−1.5927	deg
pitch	0.0038	deg
yaw	3.1399	deg
x	0.0654	m
y	−0.0781	m
z	−0.0458	m

**Table 5:** Single-lane road in neighborhood.

Class	Detection	False Detection	Total	${\bar{d}}_{\mathrm{euclidean}}$ [m]	${\bar{d}}_{\mathrm{longitudinal}}$ [m]	Valid Ranging [%]
Vehicle	82	0	82	7.59	6.82	91.4
Crosswalk Line	4	1	4	6.79	5.99	30.8
Pedestrian	4	1	5	6.25	5.54	75
Bike	1	1	2	5.67	4.70	100
Bumper Sign	1	0	1	N/A	N/A	0
Speedlimit 40	1	1	1	N/A	N/A	0

**Table 6:** Two-lane road without traffic light.

Class	Detection	False Detection	Total	${\bar{d}}_{\mathrm{euclidean}}$ [m]	${\bar{d}}_{\mathrm{longitudinal}}$ [m]	Valid Ranging [%]
Vehicle	61	0	61	9.83	9.05	75.5
Crosswalk Line	4	0	4	8.71	8.34	76.9
Pedestrian	6	0	6	7.51	7.19	14.3
Bike	1	0	2	6.55	5.09	25
Crosswalk Sign	5	0	5	14.23	13.95	3.8
Speedlimit 30	1	0	1	N/A	N/A	0
Stop Sign	1	0	1	14.36	13.79	100

**Table 7:** Three-lane road with traffic light.

Class	Detection	False Detection	Total	${\bar{d}}_{\mathrm{euclidean}}$ [m]	${\bar{d}}_{\mathrm{longitudinal}}$ [m]	Valid Ranging [%]
Vehicle	23	0	23	11.24	10.88	62.7
Red Light	2	0	2	N/A	N/A	0
Green Light	5	0	5	N/A	N/A	0
Crosswalk Line	3	0	3	8.33	7.63	66.7
Pedestrian	4	0	4	5.16	3.86	24
Bike	2	0	2	9.82	8.98	18.2
Yield Sign	2	0	2	12.88	12.59	6.67
Speedlimit 30	0	2	0	N/A	N/A	0

## Data Availability

The custom dataset created for object detector training is available at github.com/stefavpolito/yolo-automotive-dataset (accessed on 21 November 2024).
